# Maple polyphenols inhibit sortase and drastically reduce *Streptococcus mutans* biofilms

**DOI:** 10.1128/spectrum.00693-25

**Published:** 2025-08-07

**Authors:** Ahmed M. Elbakush, Oliver Trunschke, Mark Gomelsky

**Affiliations:** 1 Department of Molecular Biology, University of Wyoming 173150, Laramie, Wyoming, USA; The Ohio State University College of Dentistry, Columbus, Ohio, USA

**Keywords:** biofilm, *Streptococcus*, sortase, antibiofilm, caries, maple, polyphenol, epicatechin gallate, *Listeria*

## Abstract

Dental caries is initiated by the adherence of *Streptococcus mutans* to tooth surfaces, leading to biofilm (plaque) formation and the production of enamel-eroding acids. Sortase A (SrtA) plays a critical role in this process because it anchors many protein adhesins to the bacterial cell wall, making it a promising target for plaque prevention. Our previous research demonstrated that edible polyphenols from maple extracts—nortrachelogenin-8′-O-β-D-glucopyranoside, lariciresinol, isoscopoletin, and (-)-epicatechin gallate (ECG)—inhibit SrtA in *Listeria monocytogenes* and act as antibiofilm agents. Using *in silico* modeling based on the X-ray structure of *S. mutans* SrtA, we found that maple polyphenols, as well as a related compound, (-)-epigallocatechin gallate (EGCG), bind *S. mutans* SrtA with favorable free energy values. These polyphenols inhibited sortase activity *in vitro* and reduced *S. mutans* attachment to hydroxyapatite discs, which mimic tooth surfaces, as well as to acrylic false teeth. Among the tested compounds, ECG exhibited the strongest antibiofilm activity at a 100 µM concentration, significantly outperforming EGCG. Notably, this effect was independent of the *S. mutans* strain tested. ECG is not only present in maple extracts but is also abundant in green and black tea. Given their efficacy, safety, and natural abundance, we propose that aqueous maple extracts or ECG alone could serve as a safe, cost-effective strategy for preventing dental caries. This approach may be particularly beneficial for young children, for whom antimicrobial oral hygiene products pose toxicity concerns.

**IMPORTANCE:**

This study highlights the potential of natural compounds from maple to combat *Streptococcus mutans*, the bacteria responsible for tooth decay. We identified sortase inhibition as a key mechanism by which maple-derived polyphenols prevent the formation of biofilms on tooth-like surfaces. One of these compounds, (-)-epicatechin gallate, which is also abundant in tea leaves, emerges as a powerful and safe alternative to traditional plaque-fighting agents. Its natural abundance, affordability, and lack of toxicity make it especially promising for inclusion in oral care products like mouthwashes, offering a safer option for children who often accidentally swallow them.

## INTRODUCTION

Dental caries is one of the most common preventable human diseases that affects approximately 2.5 billion people worldwide (1). If left untreated, caries can lead to discomfort, sleep loss, dietary changes, gum abscesses, and ultimately systemic infections. The initial steps leading to dental caries involve the adherence of *Streptococcus mutans* to tooth surfaces, followed by the formation of biofilms (plaques). The high cariogenic potential of *S. mutans* has been attributed to three main factors: (i) its ability to synthesize large quantities of glucan-based exopolysaccharides, which help it adhere to tooth surfaces and form the protective extracellular matrix; (ii) its fermentation of a wide range of carbohydrates, including sucrose and glucose, into lactate and organic acids that gradually demineralize tooth enamel; and (iii) its tolerance to low pH and other environmental stressors, which facilitates its persistence in the oral cavity (2, 3). By inhibiting *S. mutans* adhesion to teeth and the formation of glucan-based exopolysaccharides, we can potentially disrupt the initial steps of dental caries development.

Approximately 50% of US children 6–9 years of age had one or more decayed, filled, or missing primary or permanent teeth (4), which suggests that current oral hygiene is inadequate, even in high-income countries. Younger children are particularly vulnerable to caries due to excessive intake of sugary and acidic drinks and food and inadequate oral hygiene. Therapeutic mouthwashes help eliminate plaque-forming bacteria, yet the antimicrobial compounds in these mouthwashes are toxic if ingested (5), which led the American Dental Association to advise against their use in children under 6 years of age (6). Because of the toxicity concern, fluoride-containing mouthwashes are also not recommended for children under six (6). In this study, we describe safe-to-ingest natural polyphenolic compounds from maple wood that prevent *S. mutans* from adhering to tooth surfaces and uncover their key mechanism of action. Among these compounds is (-)-epicatechin gallate (ECG), a safe ingredient (7) present not only in maple but also abundant in green and black tea.

This line of research emerged from our investigation of biofilms formed by the foodborne pathogen *Listeria monocytogenes* (8). We serendipitously discovered that aqueous extracts from maple wood, including maple sap and diluted maple syrup, exhibit potent antibiofilm properties against listerial biofilms. We identified several antibiofilm ingredients from maple, including lignans (nortrachelogenin-8′-O-β-D-glucopyranoside [NTG] and lariciresinol [LR]), hydroxycoumarins (scopoletin [SC] and isoscopoletin [IS]), and a catechin, ECG. Surprisingly, all these compounds were found to inhibit the same molecular target: sortase A (SrtA) (9, 10). Sortases, including SrtA, are transpeptidases that covalently anchor the C-termini of surface proteins to the peptidoglycan layer of the cell wall (11). They are widespread in bacteria belonging to the Bacillota (Firmicutes) phylum that encompasses both *Listeria* and *Streptococcus* (12). Sortases anchor many surface adhesins that facilitate bacterial attachment to abiotic surfaces and to the surface proteins of mammalian cells; therefore, the *srtA* mutants of pathogenic species, including *L. monocytogenes* and *S. mutans*, are impaired in virulence (13–16). Specifically, the *S. mutans srtA* mutant exhibits reduced binding to salivary components and diminished attachment to extracellular matrix proteins (such as collagen type I, fibronectin, and laminin). Additionally, this mutant demonstrates impaired biofilm formation and reduced bacterial coaggregation activity (17). We hypothesized that edible maple polyphenols could inhibit *S. mutans* SrtA, thereby reducing bacterial attachment to tooth surfaces and preventing caries development. The results of this study support this hypothesis, suggesting that polyphenols with antibiofilm activity may provide a promising, natural alternative for caries prevention, particularly for young children and individuals who cannot use traditional oral hygiene products containing potentially toxic antimicrobial compounds.

## MATERIALS AND METHODS

### Bacterial strains, growth conditions, and biofilm assays

The strains used in this study are listed in Table 1. Overnight liquid cultures of *S. mutans* were grown in the liquid brain heart infusion (BHI) medium (Millipore Sigma) containing 1% sucrose at 37°C under shaking (180 rpm).

**TABLE 1:** Strains and plasmids used in this study

Strains and plasmids	Description	Reference
Strains		
*Escherichia coli*		
DH10β	Strain used for plasmid maintenance	NEB
BL21 [DE3] (pLysS)	Strain used for protein overexpression	ThermoFisher
*Streptococcus mutans*		
DPC6143	Isolated from a child's saliva	18
UA159	Isolated from a child's oral cavity	19
UA140	Isolated from a human oral cavity	20
ATCC25175	NCTC 10449, isolated from a human oral cavity	ATCC
ATCC35668	LRA 28 02 81 (Clarke), used as a quality control strain	ATCC
Plasmids		
pET23a	Vector for His_6_-tagged protein overexpression	Invitrogen
SrtAΔN41-pET23a	Plasmid for StrADN41::His_6_ overexpression	This study

The effect of maple polyphenols on biofilm formation was assessed using acrylic resin false teeth (Acrylic Resin Fake Teeth, BBTO) and saliva-coated hydroxyapatite (sHA) discs (1 cm diameter; 3D Biotek, NJ). For experiments with false teeth, *S. mutans* strains were incubated in BHI broth containing 1% sucrose and the false teeth, with or without polyphenols at the 100 µM concentration, under mild shaking at 37°C for 48 h. After incubation, the false teeth were rinsed twice with distilled water, stained with 0.1% crystal violet for 15 min, rinsed three times in water, and photographed.

The effect of polyphenols on bacterial adherence to sHA was assessed as follows. The discs were coated with filter-sterilized clarified saliva (collected from volunteers) and incubated at room temperature for 1 h. A suspension of *S. mutans* cells in BHI broth containing 1% sucrose, with or without polyphenols (100 µM), was added to the wells of a 12-well cell culture plate (CellTreat Scientific Products, MA), where the sHA discs were placed. The plates were incubated under static conditions at 37°C under 5% CO_2_ for 48 h. After incubation, the discs were rinsed with sterile water to remove loosely attached bacteria. The sHA discs were then either stained with 0.1% crystal violet, as described above, or used for quantification of attached cells. To dislodge the adhered bacteria, the discs underwent mild sonication in an ultrasonic cleaner. The resulting bacterial suspension was plated onto BHI agar and incubated at 37°C for 24 h to determine the number of CFUs.

### Molecular modeling of the *S. mutans* SrtA interactions with selective polyphenols

The X-ray structure of the *S. mutans* SrtA (PDB: 4TQX) (21) was used to predict binding modes of the maple compounds using the Autodoc Vina software (22). Grid box measurements were established with “AutoDockTools-1.5.7.” Box coordinates: X: 9.116 Å, Y: 26.623 Å, and Z: −12.548 Å; box size: X: 62 Å, Y: 60 Å, and Z: 60 Å; with default spacing 0.375, mode number: 3, energy range: 5, and exhaustiveness: 8.

### Maple wood products and phytochemicals

Phytochemicals were purchased from the following suppliers: ECG and (-)-epigallocatechin gallate (EGCG) from Aobius Inc. (MA, USA), NTG, LR, and IS from Targetmol (MA, USA). Maple sap (Organic Maple Water) was purchased from Maple 3 (Canada). Maple syrup (Maple Syrup Grading Sampler, Amber) was purchased from Butternut Mountain Farm, Maple Sugar Company (VT, USA). Aqueous wood extracts were prepared by soaking maple wood chips (Camerons Smoker Wood Chips, Variety Gift Set, CO, USA) for 24 h, using 1 g of wood per 10 mL of sterile water as described earlier (9). Extracts were added at 1 mL per 10 mL culture.

### Proteins overexpression and purification

Recombinant SrtA protein was purified essentially as described earlier (23). The DNA sequence encoding SrtA (with a deletion of the first 41 amino acids, SrtAΔ41) was amplified from *S. mutans* genomic DNA using these primers: 5′-CTTTAAGAAGGAGATATACAATGTGGAATACCAATAG-3′ (forward) and 5′-GAGCTCGAATTCGGATCAAATGATATTTGATTATAG-3′ (reverse). The amplified fragment was digested with BamHI and NdeI and cloned into pET23a. The recombinant plasmid, SrtAΔ41-pET23a, was transformed into *Escherichia coli* BL21 [DE3] cells carrying pLysS (Table 1). These cells were grown in Luria-Bertani (LB) medium supplemented with ampicillin (100 mg/L) and chloramphenicol (25 mg/L) at 37°C with shaking, until A_600_, 0.6–0.8. Isopropyl β-D-1-thiogalactopyranoside was added to a final concentration of 1 mM, and the culture was incubated for an additional 12 h at room temperature. The cells were harvested by centrifugation at 4°C. The cell pellets were resuspended in buffer (pH 7.4) containing 300 mM NaCl, 50 mM NaH_2_PO_4_, 10 mM imidazole, and protease inhibitors (Protease Inhibitor Cocktail, APExBIO). Cell lysis was performed using a French press (Spectronic Instruments, NJ). The crude lysates were centrifuged at 15,000 × *g* for 10 min, and the soluble protein fraction was incubated with pre-equilibrated Co^2+^-charged resin (Talon Metal Affinity Resin, TaKaRa) at 4°C for 3 h. The resin-bound protein was loaded onto a column and washed extensively with buffer containing 25 mM imidazole. The SrtAΔ41::His_6_ protein was eluted with 250 mM imidazole. Protein purity was assessed by SDS-PAGE, and protein concentration was determined using a Bradford assay (BioRad).

### SrtA activity assays

SrtA activity was measured using a previously described protocol that monitors fluorescence changes resulting from proteolytic cleavage of a peptide substrate, Dabcyl-LPETG-Edans (AnaSpec), at the SrtA-binding site (24). Reactions (120 µL) were performed in the buffer (50 mM Tris-HCl, 150 mM NaCl, and 5 mM CaCl_2_; pH 8) containing 24 µg SrtAΔN41::His_6_ and 1 µg (8 nM) substrate. Quantification of fluorometric intensity (350 nm excitation and 520 nm emission) was done using a microplate reader (SYNERGY H4, BioTek).

### Analysis of *S. mutans* extracellular proteins

To evaluate the effect of ECG on the shedding of cell-associated proteins into the medium, *S. mutans* strains were inoculated at an initial A_600_ of 0.05 and grown for 48 h in the presence or absence of ECG. Bacterial cells were pelleted by centrifugation, and the cell-free culture supernatants were collected. Extracellular proteins were precipitated by adding (NH_4_)_2_SO_4_ to a final concentration of 200  mM, followed by overnight incubation on ice. The precipitated extracellular proteins were collected by centrifugation and resuspended in buffer. An equivalent of 2.5  mL supernatant from each sample was analyzed by SDS-PAGE.

## RESULTS

### *In silico* modeling of *S. mutans* SrtA interactions with maple polyphenols

**Fig 1:**
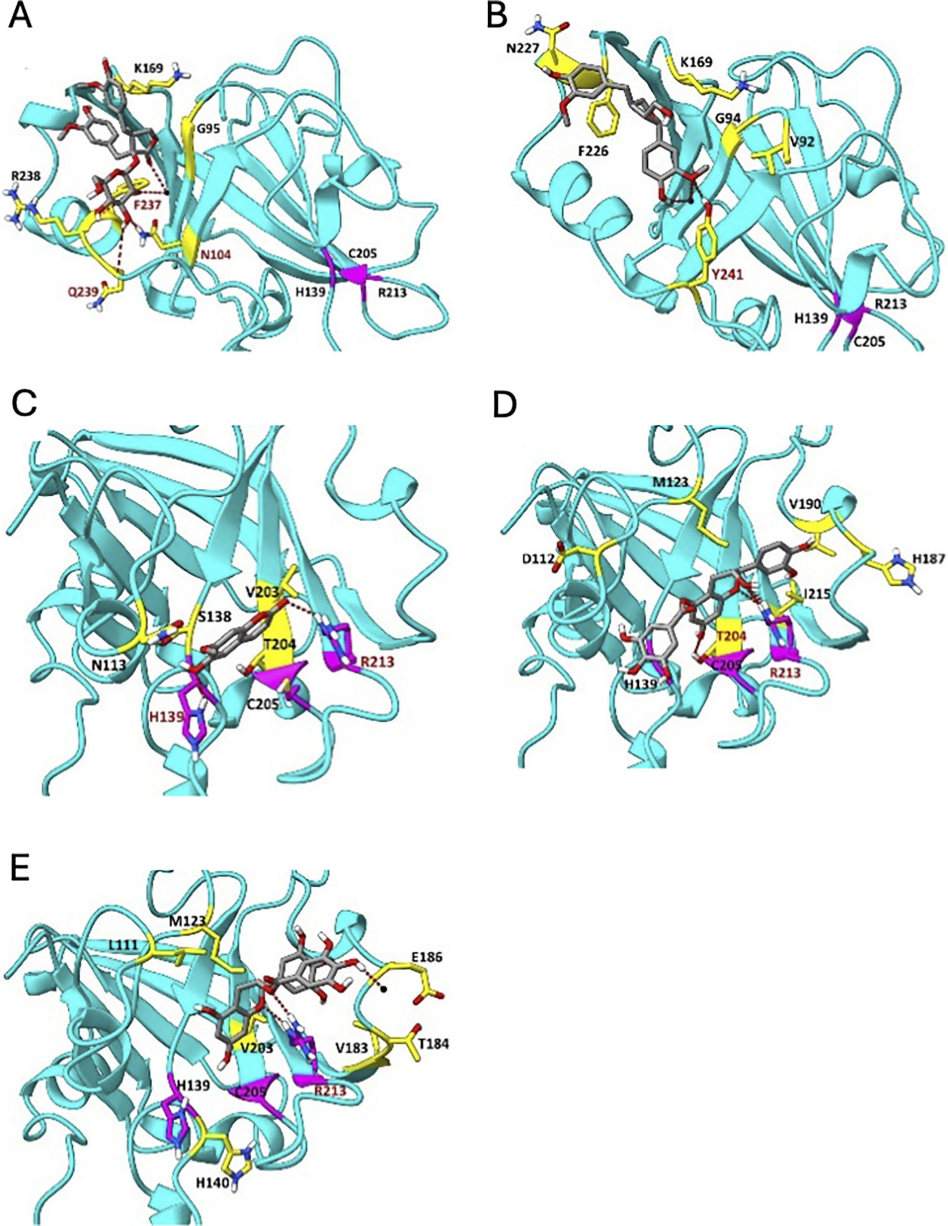
*In silico* modeling of polyphenol binding to *S. mutans* SrtA. Docking of polyphenol inhibitors with the *S. mutans* SrtA (PDB: 4TQX) using AutoDock Vina. The catalytic triad of SrtA (H139, C205, and R213) is shown in magenta. Amino acids forming H-bonds are labeled in red. Amino acids forming hydrophobic interactions are colored in yellow (except for hydrophobic interactions with H139, C205, or R213, which are shown in black). Black spheres indicate water molecules. O atoms, red; N, blue; S, yellow; H, white. Inhibitor molecules: A, NTG, B, LR; C, IS; D, ECG; E, EGCG.

To investigate whether maple polyphenols inhibit the activity of *S. mutans* SrtA, we compared primary sequences and 3D structures of the SrtA homologs from *L. monocytogenes* and *S. mutans*. Although these proteins share only moderate sequence similarity (i.e., 37% identity, 56% similarity over 89% protein length), their 3D structures are remarkably conserved (21, 24). The latter observation suggests that the inhibitors of the *L. monocytogenes* SrtA enzyme may inhibit SrtA from *S. mutans*. We therefore modeled interactions of several polyphenol inhibitors with the *S. mutans* SrtA protein using the AutoDock Vina software (22). The *in silico* models predicted that all maple-derived inhibitors of *L. monocytogenes* SrtA can bind to *S. mutans* SrtA (Fig. 1), with negative Gibbs free energies (ΔG) ranging from −5.4 to −8.1 kcal/mol. These ΔG values are comparable to those observed for binding to the *L. monocytogenes* SrtA protein (10), suggesting favorable interactions between SrtA and these polyphenols. This further strengthened our confidence that maple polyphenols may inhibit *S. mutans* sortase (Table 2).

**TABLE 2:** Predicted ΔG values (kcal/mol) for the binding of polyphenol inhibitors to SrtA

Compound	L. monocytogenes	*S. mutans*
NTG	−7.2	−6.2
LR	−6.2	−6.6
IS	−5.0	−5.4
ECG	−7.4	−8.1
EGCG	−7.7	−7.2

Among the tested maple polyphenols, ECG showed the strongest binding (ΔG, −8.1 kcal/mol). It is predicted to form H-bonds with the catalytic triad residues in SrtA, i.e., H139, C205, and R213 (Fig. 1) (21). ECG is structurally similar to EGCG, which has not been reported in maple extracts but is the most abundant polyphenol in tea. Because ECG differs from EGCG only by the absence of a single hydroxyl group, we included EGCG in our analysis. The modeling predicted that EGCG binds *S. mutans* SrtA with a strong affinity, exhibiting a ∆G of −7.2 kcal/mol, only slightly lower than that of ECG. The additional hydroxyl group in EGCG appears to alter its conformation within the SrtA binding pocket relative to ECG, potentially reducing its binding affinity (Fig. 1). The binding of NTG and LR is expected to be weaker, occurring at the periphery of the *S. mutans* SrtA catalytic pocket, suggesting that these compounds are less effective inhibitors than ECG or EGCG. While IS is predicted to interact with some catalytic residues, it has the lowest absolute ∆G value (−5.0 kcal/mol) among the tested maple polyphenols, indicating relatively weaker affinity. Overall, this computational modeling strengthens the hypothesis that maple polyphenols with inhibitory activity against *L. monocytogenes* SrtA, as well as EGCG, may also inhibit *S. mutans* SrtA.

### Maple polyphenols inhibit *S. mutans* SrtA *in vitro*

To test this hypothesis, we purified the *S. mutans* SrtA protein and measured its peptidase activity in the presence or absence of polyphenols using a fluorescently labeled peptide substrate containing the SrtA recognition motif, LPXTG, where *X* represents any amino acid (24). We overexpressed and purified the soluble fragment of the SrtA protein, omitting the 41 N-terminal residues responsible for secretion and membrane anchoring in *S. mutans*, as these residues are non-essential for catalytic activity (23).

**Fig 2:**
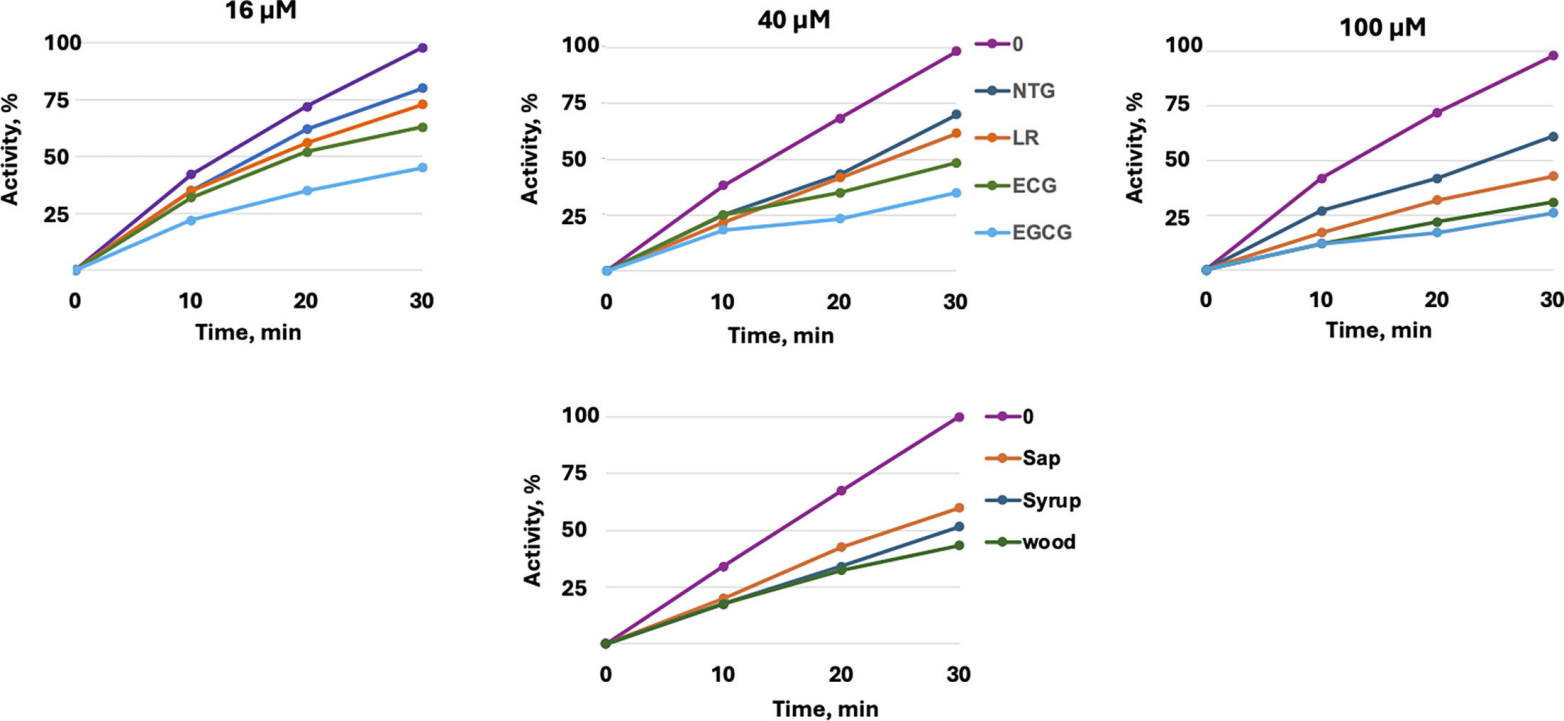
*S. mutans* SrtA inhibition by polyphenols. Peptidase activity of the purified *S. mutans* SrtAΔ41::His_6_ was tested using a fluorescently labeled LPXTG-containing peptide substrate. Activity in the absence of inhibitors, after 30 min, was assigned to 100%. *Upper panel*, sortase activity in the presence of NTG, LR, IS, ECG, and EGCG in the range of concentrations 16−100 μM. *Lower panel*, sortase activity in the presence of maple sap, diluted (1:200) maple syrup, and aqueous maple wood extract. Each data point represents the mean of three independent experiments, each performed with two technical replicates. SDs were below 17% of the mean values across all experiments.

All tested polyphenolic compounds inhibited *S. mutans* SrtA activity in a concentration-dependent manner, in the range of tested concentrations, 16−100 μM. EGCG and ECG were identified as the most potent SrtA inhibitors *in vitro*, each inhibiting SrtA by approximately 70%−75% at a 100 µM concentration, while NTG and LR exhibited weaker inhibitory effects (Fig. 2). Additionally, we tested the inhibition of *S. mutans* SrtA by maple extracts, including an aqueous maple wood extract obtained by soaking maple wood in water, as well as commercially available samples of maple sap and (1:200 diluted) maple syrup. As shown in Fig. 2, all aqueous maple extracts inhibited SrtA, which mirrors our observations of the *L. monocytogenes* SrtA (10). Encouraged by the results of the *in vitro* assays, we investigated whether the anti-SrtA activity of the maple polyphenols correlates with inhibition of the *S. mutans* biofilms.

### Maple polyphenols prevent biofilm formation by *S. mutans*

**Fig 3:**
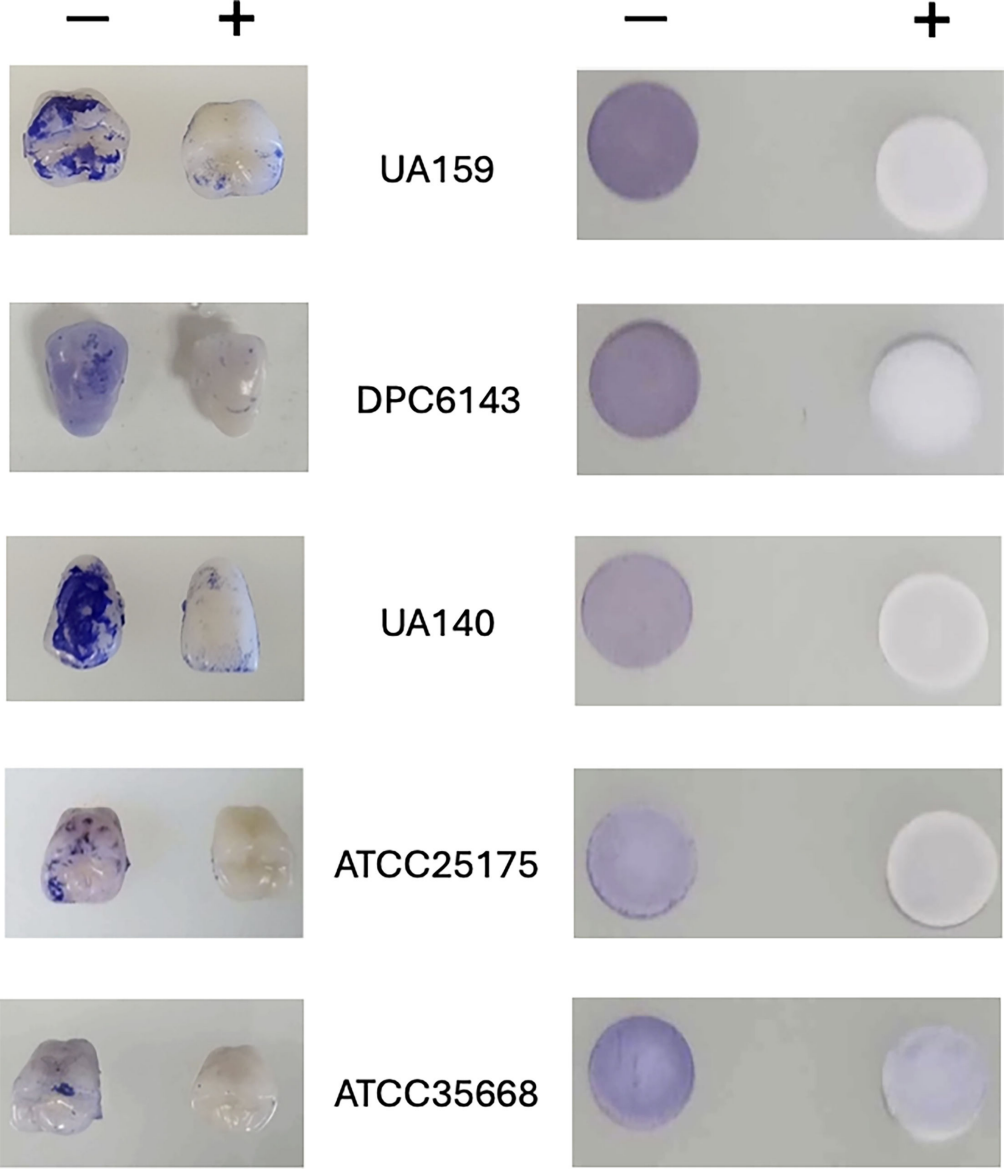
*S. mutans* biofilm inhibition by ECG. *Left panel*, acrylic resin false teeth were incubated with five different *S. mutans* strains in the absence (−) or presence (+) of ECG (100 µM, final concentration) under mild shaking at 37°C for 48 h. *Right panel*, sHA discs were incubated with various *S. mutans* strains in BHI broth containing 1% sucrose in the absence (−) or presence (+) of ECG (100 µM, final concentration) under 5% CO_2_ with no shaking at 37°C for 48 h. False teeth and sHA discs were stained with 0.1% crystal violet.

To evaluate the antibiofilm activity of maple polyphenols, we used two different surfaces relevant to dental practice, i.e., (i) hydroxyapatite discs preincubated with sterile saliva (sHA), which serve as a proxy for the tooth surfaces, and (ii) false acrylic resin teeth. To assess the universality of our observations, we slightly varied growth conditions. In all experiments, *S. mutans* strains were grown in rich (BHI) liquid medium containing 1% sucrose and 100 µM polyphenols for 48 h at 37°C. However, in the experiments with sHA, *S. mutans* was grown under static conditions in a 5% CO_2_ atmosphere, whereas in the experiments with acrylic resin teeth, *S. mutans* was incubated in ambient air with mild shaking. Biofilms formed on sHA and acrylic resin teeth were visualized using crystal violet staining. We found that biofilms formed by *S. mutans* UA159, the first tested strain, were significantly reduced in the presence of 100 µM ECG on both surfaces under both conditions (Fig. 3A and B). These results suggest that ECG’s antibiofilm activity is not limited to a specific surface or growth condition, which is consistent with the SrtA impairment as the mechanism of ECG action.

To assess whether ECG’s antibiofilm effect is strain specific, we examined biofilm formation in four additional *S. mutans* strains isolated from dental caries patients and individuals with unknown caries etiology (Table 1). As expected, biofilm formation varied across strains depending on the model surface and experimental conditions. However, in all cases, ECG markedly reduced biofilm formation (Fig. 3A and B).

**Fig 4:**
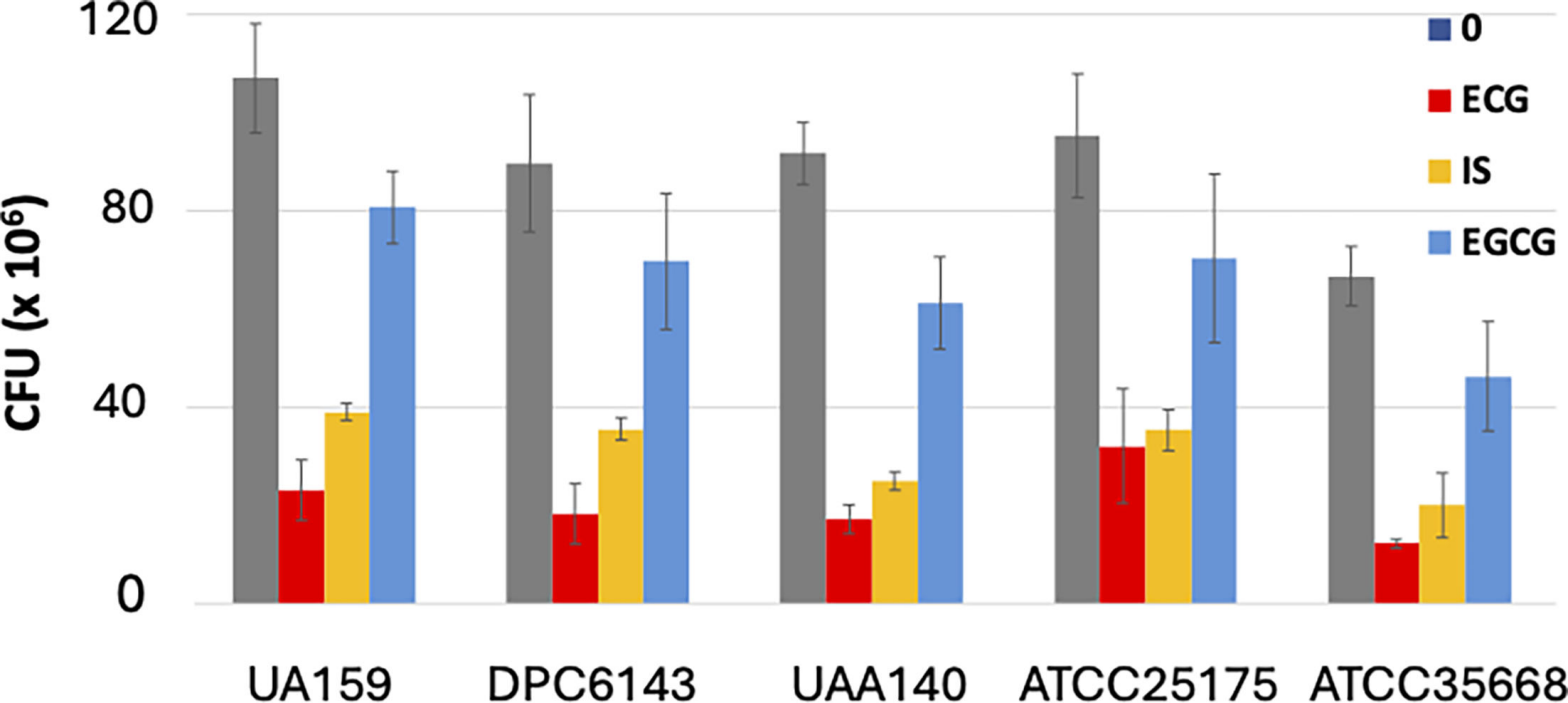
Polyphenol-mediated biofilm inhibition in various *S. mutans* strains. The *S. mutans* cells attached to sHA discs (incubated as described in Fig. 3) were detached by mild sonication. CFUs were determined by plating the dispersed bacteria onto BHI agar and incubating at 37°C for 24 h. Gray, no added polyphenols; red, ECG; yellow, IS; blue, EGCG. All polyphenols were used at a final concentration of 100 µM. Data represent average CFUs from three representative experiments, each with two technical replicates ± SD.

To quantify the antibiofilm effect of ECG and other maple polyphenols, we measured the number of *S. mutans* cells attached to sHA discs, which is expected to correlate with the enamel-destructive potential of *S. mutans*. CFUs were enumerated by incubating sHA discs with *S. mutans*, subjecting them to mild sonication to detach surface-adhered bacteria, and plating the resulting dispersed cells. As shown in Fig. 4, 100 µM ECG reduced *S. mutans* CFUs in the sHA disc assays by several fold in all strains. Notably, a brief 60 s exposure of sHA discs to 100 µM ECG, followed by a 48 h incubation with *S. mutans*, did not significantly reduce CFU counts. This suggests that ECG does not strongly or persistently bind to the sHA surface and requires sustained presence to effectively inhibit *S. mutans* biofilm formation.

Similar to ECG, 100 µM IS, another maple polyphenolic SrtA inhibitor, also decreased bacterial attachment. In contrast, 100 µM EGCG had a much smaller antibiofilm effect, despite its potent *in vitro* SrtA inhibitory activity. This lack of correlation between *in vitro* SrtA inhibition and biofilm reduction is unsurprising, as we previously observed a similar phenomenon in *L. monocytogenes* (10). The reasons for the potential discrepancy are discussed below. Finally, we tested aqueous maple wood extract that contains a mixture of polyphenol SrtA inhibitors. As expected, they exhibited strong antibiofilm activity (Fig. 4).

### ECG stimulates the release of *S. mutans* proteins into the growth medium

**Fig 5:**
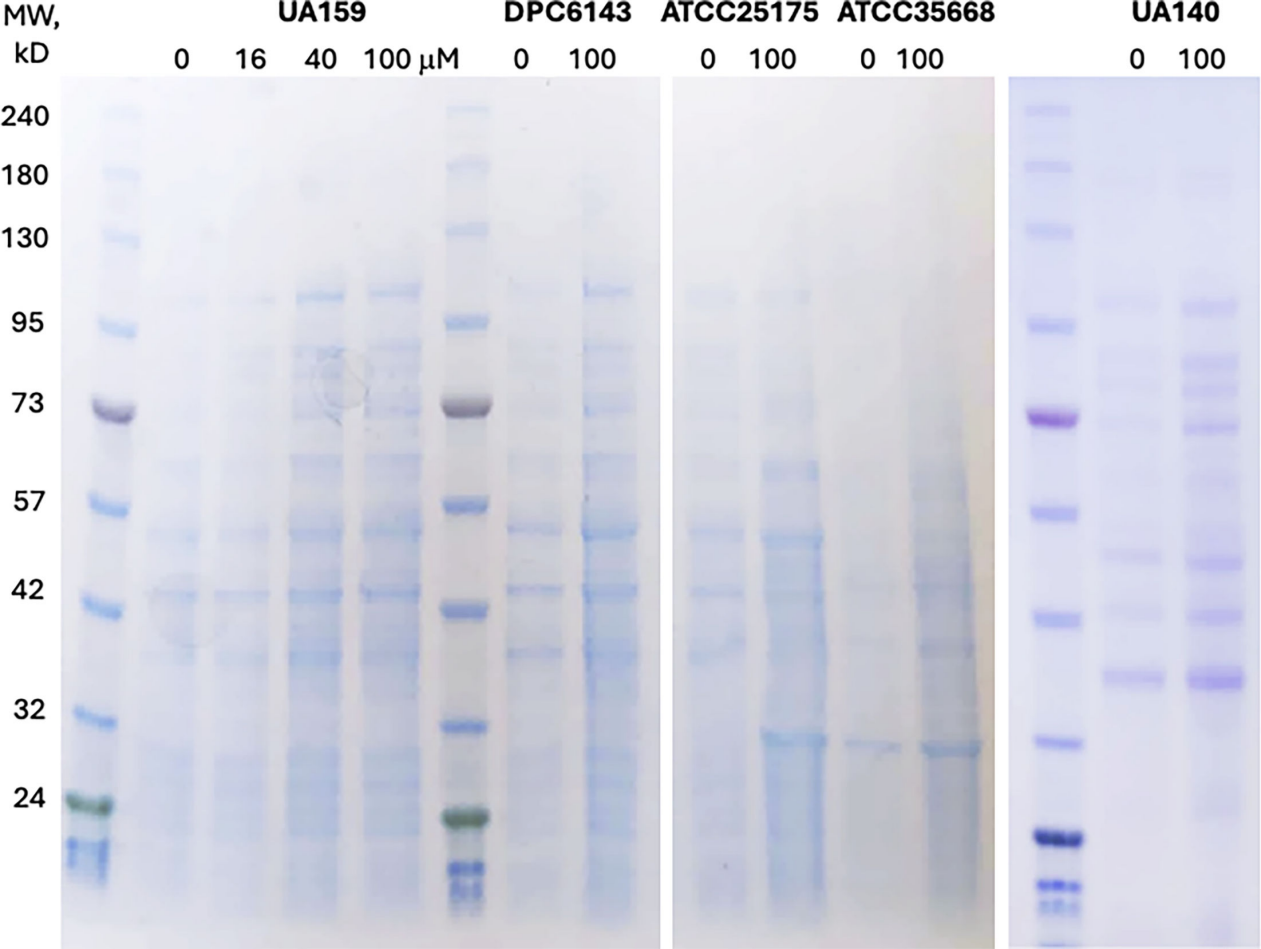
ECG-induced increase in extracellular proteins. The effect of ECG (final concentrations indicated in µM) on the shedding of cell-associated proteins into the culture medium in various *S. mutans* strains. Extracellular proteins from 48 h old cultures were precipitated with ammonium sulfate, and the protein amounts corresponding to 2.5 mL of culture supernatant were analyzed by SDS-PAGE. MW, molecular weight marker.

Since sortase inhibition is expected to prevent proper anchoring of cell wall-associated proteins, leading to their release into the medium, we tested whether ECG has this effect. *S. mutans* cultures were grown with or without 100 µM ECG, and culture supernatants were collected after removing the bacterial biomass. Extracellular proteins were precipitated from the supernatants using ammonium sulfate and analyzed by SDS-PAGE. As shown in Fig. 5, all tested *S. mutans* strains exhibited a marked increase in extracellular protein levels in the presence of ECG. Importantly, the overall protein profiles remained unchanged between ECG-treated and untreated samples, indicating that ECG did not induce cell lysis, which would have resulted in the release of cytoplasmic proteins and altered profiles. This conclusion is further supported by the observation that ECG did not inhibit *S. mutans* growth. Thus, the most parsimonious explanation is that ECG promotes the shedding of cell wall-associated proteins into the medium, consistent with its role as a sortase inhibitor.

## DISCUSSION

In this study, we demonstrated that aqueous extracts from maple wood, including sap and syrup, exhibit significant antibiofilm activity against *S. mutans*, a key pathogen involved in initiating dental caries. This activity is attributed to polyphenolic compounds present in the extracts that possess antisortase activity (Fig. 3 and 4). While sortase inhibition is likely not to be the sole mechanism underlying the antibiofilm activity of maple polyphenols, the following lines of evidence suggest that it is the dominant one. First, the phenotypes of *S. mutans srtA* mutants reported in multiple studies closely resemble those of strains treated with sortase inhibitors (15–17, 21, 25–30). Second, maple polyphenols, whether used individually or in combination (e.g., in maple extracts), inhibit biofilm formation not only in *S. mutans* but also in *L. monocytogenes* (9, 10), which differs from *S. mutans* in its protein adhesins and lacks the *S. mutans*-specific extracellular glucosyltransferases responsible for synthesizing large amounts of exopolysaccharides in the presence of sucrose (2). It is therefore highly unlikely that each polyphenol independently targets a distinct, unrelated antibiofilm target in *S. mutans* and *L. monocytogenes*. Third, extracellular protein levels in all tested *S. mutans* strains were several-fold higher in the presence of ECG, the most potent sortase inhibitor examined in this study (Fig. 5), in agreement with the hypothesis that ECG promotes the release of cell wall-associated proteins. These observations strongly support sortase inhibition as the primary mechanism.

Computational modeling revealed that maple polyphenols bind to the catalytic site of *S. mutans* SrtA, an enzyme responsible for anchoring surface proteins to the bacterial cell wall, exhibiting negative Gibbs free energy values (Fig. 1; Table 2). *In vitro* assays confirmed that the polyphenols inhibit SrtA activity in a concentration-dependent manner (Fig. 2). Given the structural and sequence similarities between SrtA proteins across different *Streptococcus* species, it is likely that these polyphenols will also inhibit sortases from other species within the genus. Sortases, located on the outer surface of the cell, are considered an “ideal” drug target (31), highlighting the importance of the ongoing search for effective inhibitors.

Our study is not the first to identify *S. mutans* SrtA inhibitors. For instance, NTG, one of the polyphenols found in maple, was previously recognized as an *S. mutans* SrtA inhibitor in a study examining bioactive compounds extracted from the roots of the medicinal plant *Pulsatilla koreana* (25). In addition, several other plant polyphenols with inhibitory activity against streptococcal SrtA have been reported (21, 26–30). Maple polyphenols offer two key advantages over previously reported inhibitors: (i) a well-established safety profile and (ii) cost-effectiveness, making them a promising alternative for practical applications. First, maple polyphenols are active at concentrations naturally present in products like maple sap, which has been safely consumed by humans for centuries. Second, these polyphenols can be extracted cost-effectively from wood processing byproducts such as shavings or chips. Furthermore, the maple syrup industry, which processes approximately one billion gallons of maple sap annually (32, 33), offers another potential source of polyphenols. However, sucrose present in the maple sap presents a challenge for the application of these polyphenols as anticaries agents.

ECG, identified as a potent maple-derived sortase inhibitor in this study, offers a promising alternative to maple polyphenol-containing solutions as a single antiplaque agent. ECG is particularly appealing because it is not only present in maple wood extracts but is found in higher concentrations in green and black tea. For instance, the ECG content in a typical 250 mL cup of green tea varies from 3 to 27 mg (34), i.e., from 40 to 360 µM, which is in the same range as the 100 µM concentration used in the biofilm inhibition experiments (Fig. 3 and 4). ECG’s higher solubility in water, compared to other known SrtA inhibitors like curcumin or morin (35, 36), further enhances its potential as an effective and easily formulable anticaries agent.

Tea, particularly green tea, has long been recognized for its potential in caries prevention, with its anticaries effects traditionally attributed to EGCG, the most abundant polyphenol in tea (37). EGCG exhibits bactericidal activity against *S. mutans*, though this effect is highly dependent on the growth medium (38). In the rich media containing sucrose, such as the media used in our experiments, EGCG displays no antibacterial activity at 100 µM, a finding consistent with previous studies (39). However, EGCG does exhibit inhibitory effects on *S. mutans* glucosyltransferases, enzymes responsible for the synthesis of insoluble exopolysaccharides, a key component in biofilm formation (40, 41). Our finding that EGCG inhibits *S. mutans* SrtA, a key factor contributing to its antibiofilm activity, is consistent with previous reports that showed that EGCG also inhibits SrtA in other *Streptococcus* species, including *Streptococcus pneumoniae* (42) and *Streptococcus suis* (43). A key finding of this study is that ECG, an abundant polyphenol found in tea, strongly inhibits *S. mutans* SrtA *in vitro* (Fig. 2) and exhibits significantly greater antibiofilm activity against *S. mutans* than EGCG (Fig. 4).

The discrepancy in antisortase and antibiofilm activities between ECG and EGCG warrants further exploration. One plausible explanation lies in the differential affinity of these two polyphenols for lipid bilayers. ECG has been shown to interact more strongly with lipid membranes than EGCG (44), which could result in higher local concentrations of ECG within the bacterial membrane. This enhanced concentration might facilitate better targeting of membrane-bound SrtA, thereby potentiating its antibiofilm activity. Furthermore, in a related Bacillota species, *Staphylococcus aureus*, ECG induces perturbation in lipid bilayers, resulting in a broader disruption in membrane- and cell-wall-associated proteins (45).

The potent antibiofilm activity of ECG at the 100 µM concentration makes it a promising candidate for incorporation into dental care products. ECG poses no risk if accidentally ingested, which makes it suitable for younger children who are prone to swallowing mouthwash. Furthermore, unlike tea, ECG solutions are colorless and do not stain tooth surfaces, making them an attractive alternative for everyday oral hygiene products.

## Data Availability

The raw data supporting the conclusions of this article will be made available by the authors, without undue reservation.
